# Late initiation of antenatal care and associated factors among pregnant women attending antenatal clinic at Hiwot Fana Comprehensive Specialized Hospital, Eastern Ethiopia: a cross-sectional study

**DOI:** 10.3389/fgwh.2024.1431876

**Published:** 2024-11-27

**Authors:** Bayisa Dibaba, Miressa Bekena, Tariku Dingeta, Eshetu Refisa, Habtamu Bekele, Shambel Nigussie, Eyobel Amentie

**Affiliations:** ^1^ School of Medicine, College of Health and Medical Sciences, Haramaya University, Harar, Ethiopia; ^2^ School of Public Health, College of Health and Medical Sciences, Haramaya University, Harar, Ethiopia; ^3^ School of Midwifery, College of Health and Medical Sciences, Haramaya University, Harar, Ethiopia; ^4^ School of Pharmacy, College of Health and Medical Sciences, Haramaya University, Harar, Ethiopia

**Keywords:** late initiation, pregnant women, antenatal care, Harar, Ethiopia

## Abstract

**Background:**

Late initiation of antenatal care (ANC) is a major public health concern. If women initiate ANC late, they do not get adequate care, reducing the chances of early detection of pregnancy-related complications. However, there is a lack of data related to the initiation of antenatal care in the study area.

**Objectives:**

To assess the prevalence of late initiation of antenatal care and identify associated factors among pregnant women attending antenatal care at Hiwot Fana Comprehensive Specialized Hospital, Eastern Ethiopia.

**Methods:**

An institutional-based cross-sectional study design was conducted among 454 pregnant women. All eligible participants during the study period were included. Data were collected via face-to-face interviews, were entered into Epi Data version 4.3, and analyzed using SPSS version 22 software. The results are presented using texts and tables. Logistic regression with the 95% confidence interval (CI) was used to identify factors associated with the late initiation of antenatal care. Statistical significance was declared at *P* < 0.05.

**Results:**

The prevalence of late-initiation antenatal care in this study was 59.5% (95% CI 54.6–63.4). Age range of 30–34 years [adjusted odds ratio (AOR) 2.7, 95% CI 1.69–13.1] and age ≥35 years (AOR 4.2, 95% CI 1.92–9.84), rural residency (AOR 2.92, 95% CI 1.59–5.39), unplanned pregnancy (AOR 2.3, 95% CI 1.35–8.11), inability to make the decision to start ANC (AOR 2.14, 95% CI 1.18–3.89), multigravidity (AOR 1.9, 95% CI 1.13–12.4), wrong perception on the time of antenatal care initiation (AOR 5.8, 95% CI 3.71–9.34), lack of previous ANC experience (AOR 2.01, 95% CI 1.14–5.81), and more than 10 km distance from the hospital (AOR 1.36, 95% CI 0.62–2.95) were associated with late initiation antenatal care in this study.

**Conclusion:**

More than half of the study participants were initiated into ANC after the 12th week of gestation. Moreover, rural residence, unplanned pregnancy, age ≥30 years, lack of previous antenatal care, inability to make decisions, and wrong perception on the time of initiation of ANC were found to be associated with late initiation of ANC. Educating women and involving partners and family members in discussions about ANC were recommended to build a supportive environment for pregnant mothers.

## Introduction

Antenatal care (ANC) is essential to ensure the best health conditions for both mother and baby during pregnancy. It is aimed at risk identification, prevention, and management of pregnancy-related or concurrent diseases and health promotion (1). ANC also reduces maternal and perinatal morbidity and mortality both directly, through the detection and treatment of pregnancy-related complications, and indirectly, through the identification of women at increased risk of developing complications during labor and delivery (2). As a result, the timing of the initiation of the first antenatal care visit is paramount for ensuring optimal care and health outcomes for women and children (3).

An early ANC visit offers the opportunity to deliver preventive measures, screenings, and tests that are most effective in the early stages of pregnancy. Moreover, early initiation of ANC enables accurate assessment of gestational age to allow for the precise treatment of preterm labor, screening for genetic and congenital disorders, and timely folic acid supplementation to reduce the risk of neural tube defects. On the other hand, early initiation of ANC allows for timely screening and treatment of iron deficiency anemia as well as the identification and management of HIV, syphilis, and other sexually transmitted infections (4). In addition, early initiation of ANC can potentially capture non-communicable diseases, such as diabetes mellitus, hypertension, and chronic renal disease, and provide guidance on modifiable lifestyle risks, such as smoking, alcohol consumption, drug abuse, obesity, malnutrition, and occupational exposures (4).

Despite all interventions, over 300,000 women die each year globally from complications during pregnancy and childbirth (4). A recent maternal mortality report highlighted that approximately 800 women die daily from preventable pregnancy- and childbirth-related causes. This means that a single woman dies every 2 min, with 86% of these deaths in low- and middle-income countries (LMICs) (4).

Several studies have shown the associated factors that contributed to late-initiation antenatal care. These include maternal education, maternal age, pregnancy goal, wealth status/income, parity, a history of abortion or stillbirth, marital status, media exposure, distance from the health facility, residence, and health insurance (5–10).

To the best of the authors’ knowledge, no objective evidence currently demonstrates the magnitude of late initiation of antenatal care and its associated factors, especially based on the most recent definition of early initiation ANC in our study area.

As a result, the aim of the present study was to fill that gap by assessing the extent of late initiation of antenatal care and its associated factors among pregnant women who visit antenatal clinic at Hiwot Fana Comprehensive Specialized Hospital (HFCSH).

## Methods and materials

### Study area and period

This study was conducted at HFCSH in Harar town between 1 August and 30 October 2021. Harar, the capital city of Harari regional state, is located 525 km east of Addis Ababa, the capital city of Ethiopia. Based on the 2007 Census conducted by the Central Statistical Agency of Ethiopia (CSA), Harar, has a total population of 183,415, of whom 92,316 were men and 91,099 were women. There are five hospitals in Harar: one university teaching hospital, one regional hospital, one federal police hospital, one fistula hospital, and one private hospital. In addition, there are 8 health centers, 29 private clinics, 26 health posts, and 1 regional laboratory. HFCSH is one of the hospitals in the city administered under Haramaya University and serves as a tertiary teaching hospital. It serves as the referral hospital for Harari regional state and East Hararghe zone. It has four departments (medicine, surgery, pediatrics, and gynecology and obstetrics) with 33, 42, 50, and 60 beds, respectively, and five other departments (psychiatry, emergency and critical care, anesthesiology, dermatology, and orthopedics). There are also well-established maternal and child health (MCH) services that provide antenatal care, family planning, and vaccination in the hospital. The MCH service provides antenatal care for approximately 596 women every 3 months and 194 women per month based on the hospital health management and information system (HMIS) data. In addition, the hospital provides other services through an oncology center, a dental clinic, radiology, pathology, and a chronic follow-up clinic.

### Study design

A cross-sectional study design was conducted.

### Source population

All pregnant women who initiated antenatal care at HFCSH were considered the source population.

### Study population

All pregnant women who initiated antenatal care at HFCSH during the study period were considered the study population.

### Eligibility criteria

#### Inclusion criteria

All pregnant women who initiated antenatal care at HFCSH during the study period were included.

#### Exclusion criteria

Pregnant women who were severely ill and those in labor were excluded from the study.

### Sample size determination and sampling technique

The sample size was originally calculated for each objective by using a single population proportion formula, by using the prevalence of the late initiation of antenatal care of 81.5% (11), and a double population proportion formula, with the assumptions of 95% confidence interval (CI), 5% error margin, 80% power, and an exposed to unexposed ratio of 1:1, based on the associated factors of late initiation of antenatal care, such as distance from health facilities, age, and family size, using information from Epi Data (12–14). We then took the largest sample size for our study, which was 462 (Table 1).

**Table 1:** Sample size calculation based on associated factors using Epi Data info.

Factor	Assumption CI: 95 Power: 80% Ratio: 1:1	Sample size	Reference
Distance from health facility	Exposed: 53.42 Unexposed: 40	**462**	(12)
Age	Exposed: 40 Unexposed: 54.13	418	(13)
Family size	Exposed: 40 Unexposed: 54.55	396	(14)

Bold Indicates the accepted sample size.

During the pilot study and review of previous medical records, the authors observed a low weekly attendance of women initiating ANC at the antenatal clinic in the study area. As a result, to obtain an adequate sample size, they adopted a total population sampling method, given that the expected number of respondents was less than the estimated sample size. The authors selected study participants based on the predefined eligibility/inclusion criteria relevant to the study's objective and population. Based on this, all pregnant women who initiated antenatal care and consented to participate during the study period were included until the required sample size was achieved.

### Data collection tools and techniques

A questionnaire was developed based on studies conducted in Ethiopia and Africa to assess the late initiation of antenatal care and its associated factors (1, 4, 15) and was adapted to suit the local context. The questionnaire was originally prepared in English and then translated into local languages. Topics consist of sociodemographic characteristics, health facility-related factors, and obstetric factors. Data collectors and supervisors received 2 days of training on the study’s objectives, response confidentiality, questionnaire content, and how they approach study participants. The training was conducted by the principal investigator.

### Study variables

In this study, the late initiation of antenatal care was the dependent variable while the independent variables were sociodemographic factors, obstetric factors, pre-existing medical disorders, and health facility-related factors.

### Data quality assurance

Before the actual data collection, a pre-test was conducted at Jugel Hospital with 5% of the sample size to assess the reliability, clarity, sequence, consistency, and understandability of the questionnaire. Based on the feedback received, necessary revisions were made to improve the quality of the final tool. Trained BSc midwives were responsible coordinating the data collection process and supervising the data collectors.

A 2-day training was provided for both data collectors and supervisors, covering the study objectives, data collection tool, methods of data collection, procedures for checking the completeness of data, and how to maintain confidentiality. Proper coding and categorization of data were maintained to ensure quality for analysis. All data were checked for completeness, accuracy, clarity, and consistency by principal investigator and supervisors before entering the data into the software. Double data entry was performed to validate the data and the entries were compared to the original data to ensure accuracy.

### Data processing and analysis

All questionnaires were examined for completeness and consistency during data management, storage, and analysis. Any errors identified during data entry were corrected promptly, and data editing and cleaning were given due attention.

Data entry, data cleaning, and coding were performed using Epi Data version 4.6 and then exported to SPSS version 22.0 for statistical analysis. Analytical statistics were carried out, and the results were presented using text, frequency tables, and graphs. The associations between dependent and independent variables were assessed using bivariate logistic regression analysis. Variables with *p*-values <0.25 in the bivariable analysis were entered into the multivariable analysis model. A multivariable logistic regression analysis model was built to assess the influence of possible potential confounding variables and the strength of association. The adjusted ORs and their 95% CIs were calculated to determine the statistically significant associations between independent and dependent variables. *p* < 0.05 was considered statistically significant.

### Operational definitions

Early initiation for ANC is defined as a pregnant woman initiating antenatal care in the first 12 weeks of gestation (1). Late initiation for ANC is defined as a pregnant woman initiating antenatal care after 12 weeks of gestation (1).

## Results

### Sociodemographic characteristics

In this study, a total of 454 pregnant women who presented to an antenatal clinic for the first time during the study period were interviewed. The mean age of those study participants was 25.27 ± 5.107 years. Approximately 140 (30.8%) participants were within the age group of 20–24 years. Out of 454 participants, 279 (61.5%) lived in urban areas, 304 (67.0%) were housewives, and 423 (93.1%) were married (Table 2).

**Table 2:** Sociodemographic characteristics of women attending their first ANC visit at HFCSH between 1 August and 30 October 2021 in Harar, Eastern Ethiopia.

Variables	Category	Frequency	%
Age	15–19	61	13.4
	20–24	129	28.5
	25–29	110	24.2
	30–34	98	21.6
	≥35	56	12.3
Residence	Urban	279	61.5
	Rural	175	38.5
Marital status	Single	13	2.8
	Married	423	93.1
	Divorced and widowed	18	3.9
Religion	Orthodox	137	30.2
	Muslim	253	55.7
	Protestant	55	12.1
	Catholic	9	2.0
Educational status	Unable to read and write	134	29.5
	Able to read and write	75	16.5
	Primary education	61	13.4
	Secondary education	75	16.5
	Tertiary education	109	24.0
Occupation	Housewife	304	67.0
	Self- employee	62	13.7
	Gov’t employee	82	18.1
	NGOs employee	4	0.9
	Student	2	0.4
Husband education	Unable to read and write	87	19.2
	Able to read and write	46	10.1
	Primary education	55	12.1
	Secondary education	79	17.4
	Tertiary education	187	41.2
Husband Occupation	Farmer	115	25.3
	Self- employee	168	37.0
	Gov’t employee	148	32.6
	NGOs employee	12	2.6
	Student	11	2.4

### Prevalence of late initiation of antenatal care

Among the 454 pregnant women who participated in this study, 270 (59.5%) had a late initiation of antenatal care. Out of the 253 (55.7%) women who had previous ANC experience, 70 (27.7%) were not advised when to start ANC by a health professional.

### Obstetric, medical, and other related characteristics

Of the 454 women, 300 (66.1%) were multigravida and 154 (33.9%) were primigravida. Approximately 51 (11.2%) women had three or more living children. Among the total study participants, 285 (62.8%) respondents learned of their pregnancy from a urine human chorionic gonadotropin (HCG) test. Approximately 34 (7.5%) participants had medical complications, of which hypertension and diabetes mellitus accounted for 13 (2.9%) and 7 (1.5%), respectively. Of the respondents, 409 (90.1%) women planned for pregnancy and 253 (55.7%) had previous ANC experience. Regarding health facility factors, 87 (19.2%) women traveled more than 2 h to reach the hospital, and 98 (21.6%) traveled more than 10 km to the study area (Tables 3, 4).

**Table 3:** Obstetric history of women attending their first ANC visit at HFCSH between 1 August and 30 October 2021 in Harar, Eastern Ethiopia.

Variables	Category	Frequency	%
GA at first visit (weeks)	≤12	184	40.5
	13–27	175	38.5
	≥28	95	20.9
Planned pregnancy	Yes	390	85.9
	No	64	14.1
Wanted pregnancy	Yes	445	98.0
	No	9	2.0
Gravidity	Primi	154	33.9
	Multi	300	66.1
Parity	Nullipara	175	38.5
	Para one and above	279	61.5
Number of alive children	No children	178	39.2
	1–2	225	49.6
	≥3	51	11.2
Means of pregnancy recognition	Urine test	285	62.8
	Missed menses	142	31.3
	Ultrasound	27	5.9
Illness during current pregnancy	Yes	37	8.1
	No	417	91.9
Presence bad obstetric history	Yes	18	4.0
	No	436	96.0

**Table 4:** Medical and other related characteristics of women attending their first ANC visit at HFCSH between 1 August and 30 October 2021 in Harar, Eastern Ethiopia.

Variables	Category	Frequency	%
Medical complication/comorbidity	Yes	34	7.5
	No	420	92.5
Specific medical complications	DM	7	1.5
	HTN	13	2.9
	Chronic renal disease	2	0.4
	Epilepsy	5	1.1
	HIV	1	0.2
	Asthma/COPD	4	0.9
	Cardiac	1	0.2
	Other	1	0.2
Ability of decision to attend ANC	Yes	278	61.2
	No	176	38.8
Decision maker to start ANC	Husband	74	45.1
	Mother	50	30.5
	Colleague	9	5.5
	Health professional	29	17.8
	Other	2	1.2
Perceived right time of initiation of ANC	Before 12 weeks	250	55.1
	After 12 weeks	204	44.9
Previous information about the time of initiation of ANC?	Yes	292	64.3
	No	162	35.7
Distance of residence from study area (HFCSH) (km)	<10	356	78.4
	>10	98	21.6

DM, diabetes mellitus; HTN, hypertension; COPD, chronic obstructive pulmunary disease.

### Factors associated with late ANC initiation

Binary logistic regression was used to identify factors associated with late initiation ANC. All the independent variables with a *p*-value <0.25 in bivariable logistic regression analysis, such as age, residence, pregnancy planning, presence of medical complications, gravidity, number of living children, perceived right time to initiate ANC, previous ANC experience, and distance from the hospital were taken to multivariable logistic regression to control for confounding factors.

In the multivariable logistic regression, age range of 30–34 years [adjusted odds ratio (AOR) 2.7, 95% CI 1.69–13.1], age ≥ 35 years (AOR 4.2, 95% CI 1.92–9.84), rural residency (AOR 2.92, 95% CI 1.59–5.39), unplanned pregnancy (AOR 2.3, 95% CI 1.35–8.11), inability to make the decision to start ANC (AOR 2.14, 95% CI 1.18–3.89), multigravidity (AOR 1.9, 95% CI 1.13–12.4), wrong perception about the time of antenatal care initiation (AOR 5.8, 95% CI 3.71–9.34), lack of previous ANC experience (AOR 2.01, 95% CI 1.14–5.81), and more than 10 km distance from the hospital (AOR 1.36, 95% CI 0.62–2.95) were significantly associated with late initiation antenatal care in this study (Table 5).

**Table 5:** Multivariable analysis of associated factors of late initiation of antenatal care among pregnant women attending their first ANC visit at HFCSH between 1 August and 30 October 2021 in Harar, Eastern Ethiopia.

Variable	Category	Late initiation of ANC		COR (95% CI)	AOR (95% CI)	*P*-value
		Yes (%)	No (%)			
Age (years)	15–19	30 (49.2)	31 (50.8)	1	1	
	20–24	63 (48.8)	66 (51.2)	0.9 (0.53–1.81)	0.71 (0.44–2.65)	0.860
	25–29	56 (50.9)	54 (49.1)	1.07 (0.57–2.0)	0.73 (0.57–3.58)	0.441
	30–34	74 (75.5)	24 (24.5)	3.1 (1.61–6.29)	2.7 (1.69–13.1)	0.003*
	≥35	47 (83.9)	9 (16.1)	5.3 (2.25–12.9)	4.2 (1.92–9.84)	0.002*
Residence	Urban	129 (46.2)	150 (53.8)	1	1	
	Rural	141 (80.6)	34 (19.4)	4.8 (3.09–7.5)	2.92 (1.58–5.39)	0.001*
Planned pregnancy	Yes	219 (56.2)	171 (43.8)	1	1	
	No	51 (79.7)	13 (20.3)	3.06 (1.61–5.81)	2.3 (1.35–8.11)	0.009*
Existing medical problem	Yes	16 (43.2)	21 (56.8)	1	1	
	No	254 (60.9)	163 (39.1)	2.04 (1.03–4.03)	1.83 (0.81–7.85)	0.064
Decision-making	Yes	137 (49.3)	141 (50.7)	1	1	
	No	133 (75.6)	43 (24.4)	3.18 (2.09–4.8)	2.14 (1.18–3.89)	0.012*
Gravidity	Primi	70 (45.5)	84 (54.5)	1	1	
	Multi	200 (66.7)	100 (33.3)	2.4 (1.61–3.57)	1.9 (1.13–12.4)	0.031*
Number of alive children	0	98 (55.1)	80 (44.9)	1	1	
	1–2	132 (58.7)	93 (41.3)	1.1 (0.77–1.7)	0.81 (0.61–4.58)	0.312
	≥3	40 (78.4)	11 (21.6)	2.9 (1.4– 6.15)	1.47 (0.41–5.39)	0.539
Previous ANC experience	Yes	129 (51.0)	124 (49)	1	1	
	No	141 (70.1)	60 (29.9)	2.25 (1.51–3.3)	2.01 (1.14–5.81)	0.001*
Perceived right time to initiate ANC in weeks	<12	98 (39.2)	152 (60.8)	1	1	
	>12	172 (84.3)	32 (15.7)	8.3 (4.2–13.1)	5.8 (3.71–9.34)	0.001*
Distance from study area (HFCSH) (km)	<10	188 (52.8)	168 (47.2)	1	1	
	>10	82 (83.7)	16 (16.3)	4.58 (2.57–8.13)	1.36 (0.62–2.95)	0.442

COR, crude odds ratio.

*Statistically significant association with *P* < 0.05.

## Discussion

In this study, the proportion of late ANC initiation among pregnant mothers was 59.5% (95% CI 54.6–63.4). Women aged 30 years or older, living in rural areas, unplanned pregnancy, not making decisions by themselves to initiate ANC, lack of ANC experience, and wrong perception about the time of ANC initiation were associated with late initiation of ANC.

The present study revealed that more than half of the respondents (59.5%) initiated ANC after the recommended time. This result is consistent with other studies conducted in Burkina Faso (16) and Woldia, North Wollo, Ethiopia, in 2020 (17). This highlights the ongoing issue of inadequate ANC utilization among pregnant women in LMICs like Ethiopia, which could hinder efforts to meet the Sustainable Development Goal of reducing maternal and perinatal morbidity and mortality.

On the other hand, the finding from this study regarding the magnitude of late initiation of antenatal care was higher than those reported in studies conducted in Addis Ababa (18) and Debre Markos town, northern Ethiopia, in 2018 (19) and Addis Zemen, Northwest Gondar, in 2019 (12), which showed rates of 42%, 33.4%, and 52.5%, respectively. This could be because Addis Ababa, being the capital of the country, likely has better awareness and access to health services compared with other parts of the country. Another reason could be the difference in the classification of outcomes, as the study in Debre Markos considered mothers to be late for initiation of ANC if they presented after 16 weeks of gestation, whereas our study defined late initiation as occurring after 12 weeks of gestation, in line with the latest WHO classification. Therefore, the authors recommend that future researchers and policymakers adopt the WHO classification system (1).

This study showed that the magnitude of late initiation is lower than that found in studies conducted in Ambo, Ethiopia (86.8%) (20), Kembata Tembaro Zone, Ethiopia (68%) (21), East Welega, Ethiopia (81.6%) (11), Tanzania (70.4%) (15), Zambia (72%) (22), and Nigeria (82.6%) (23). This could be related to socio-cultural differences in the study population.

According to this study, mothers with unplanned pregnancies had 2.3 times higher odds of initiating ANC late compared to mothers with planned pregnancies. This finding is consistent with a study from Addis Zemen (12), where mothers with unintended pregnancies were more likely to book ANC late than those with planned pregnancies. The possible reasons for this could include a lack of support from partners or families, which may reduce their health-seeking behavior. In addition, women with unplanned pregnancies may initially attempt to deny their pregnancies, to themselves and to others, leading to delayed motivation for early ANC. Furthermore, unplanned pregnancies are also associated with socio-cultural determinants of health-seeking behaviors, sexual violence, and barriers to accessing care, all of which may contribute to the late initiation of ANC.

On the other hand, this study showed that those women who perceived that ANC initiation should occur after 12 weeks were 5.8 times more likely to initiate ANC late. This finding is consistent with studies from Debre Markos town (19), Adigrat town (24), Addis Ababa (18), and Malawi (25). The lack of effective community health programs to educate women about the importance of early antenatal visits, lack of information on the purpose of early antenatal care, and the tendency to stick to the usual wrong practices and perceptions about the timing of ANC could explain this misperception (26).

This study also showed that women with no previous ANC experience were more likely to start antenatal care late, which a likelihood two times greater than those with prior ANC experience (AOR 2.01, 95% CI 1.14–5.81). This is consistent with the study in Kembata Tembaro (27). This could be due to the information received from healthcare providers about the appropriate time for initiating ANC, which contributes to the knowledge and practice of early initiation.

### Strength and limitations of the study

Being a prospective face-to-face interview of participants and the outcome classification based on the recently recommended WHO ANC contact guidelines were strengths of this study. However, there are limitations. The study was conducted at a single tertiary hospital, which may not accurately reflect the actual rate of late initiation antenatal care in the region, as many clients may visit nearby health facilities. In addition, the total population sampling method may limit the generalizability of the results to the entire population.

## Conclusion

This study revealed that nearly two-thirds of women initiated their first antenatal care visit after 12 weeks of gestation. More than half of the study participants began antenatal care later than the 12th week. In addition, factors such as rural residence, unplanned pregnancy, age ≥30 years, lack of previous antenatal care, inability to make decision, and wrong perception about the time of antenatal care initiation were found to be associated with late initiation.

Educating women about the recommended timing for their first ANC visit, involving partners and family members in discussions about pregnancy planning, and improving women’s decision-making abilities can help create a supportive environment for expectant mothers to seek timely antenatal care.

## Data Availability

The raw data supporting the conclusions of this article will be made available by the authors, without undue reservation.
